# Association of Chronic Pain With Participation in Motor Skill Activities in Children With Cerebral Palsy

**DOI:** 10.1001/jamanetworkopen.2021.15970

**Published:** 2021-07-07

**Authors:** Haresh D. Rochani, Christopher M. Modlesky, Li Li, Barbara Weissman, Joshua Vova, Gavin Colquitt

**Affiliations:** 1 Karl E. Peace Center for Biostatistics, Jiann-Ping Hsu College of Public Health, Georgia Southern University, Statesboro; 2 Department of Kinesiology, University of Georgia, Athens; 3 Department of Health Sciences and Kinesiology, Georgia Southern University, Statesboro; 4 Department of Neurology, Children’s Healthcare of Atlanta, Atlanta, Georgia; 5 Department of Pediatrics, Emory University School of Medicine, Atlanta, Georgia; 6 Department of Physical Medicine and Rehabilitation, Children’s Healthcare of Atlanta, Atlanta, Georgia

## Abstract

This cross-sectional study examines the association between pain and activities requiring motor skill performance among a nationally representative sample of US children with cerebral palsy.

## Introduction

Many children with cerebral palsy (CP) experience the secondary condition of pain, which is problematic because children with CP face barriers to participation in motor skill activities.^1,2^ Motor performance in activities of daily living among children with CP is markedly lower than that among their age-matched peers.^3^ Because of the potential effect of pain on children with CP, we sought to examine the association between pain and activities requiring motor skill performance (ie, difficulty dressing or bathing and participation in sport) among a nationally representative sample of US children with CP.

## Methods

This cross-sectional study follows the Strengthening the Reporting of Observational Studies in Epidemiology (STROBE) reporting guideline for observational studies.^4^ The Georgia Southern University institutional review board approved this study under exempt status and with a waiver of informed consent because data were publicly available and deidentified, in accordance with 45 CFR §46.

Data were extracted from the 2017 to 2018 National Survey of Children’s Health, which is a parent-reported national survey of the health of children aged 0 to 17 years in the US.^5^ We examined responses from 151 participants aged 6 to 17 years with a medical diagnosis of CP and 9177 typically developing children (TDC). Prevalence estimates were compared between children with CP and TDC using a χ^2^ test. We used binary logistic regression models to examine associations among CP, pain, difficulty dressing or bathing, and participation in sport while controlling for age, sex, and race. Race was assessed as part of the national survey to address demographic variables related to child health and was identified by participants through survey responses. Statistical significance was set at *P* < .05, and all hypothesis tests were 2 sided. A multiple imputation method was adopted to handle missing data. We used SAS statistical software version 9.4 (SAS Institute) to analyze the data. Data analysis was performed from April 2020 to February 2021.

## Results

Table 1 provides descriptive characteristics of the 151 children with CP (mean [SD] age, 12.124 [3.42] years; 91 boys [60.3%]) and 9177 TDC (mean [SD] age, 12.27 [3.52] years; 4446 boys [48.5%]). Compared with TDC, children with CP were much more likely to have difficulty dressing or bathing (83 participants [56.9%] vs 15 participants [0.2%]) and less than one-half as likely to participate in a sports activity (43 participants [30.1%] vs 5998 participants [66.3%]). Chronic pain was also 4.5 times more prevalent in children with CP (50 participants [33.1%] vs 665 participants [7.3%]). In addition, there was a significant difference in household incomes between those with CP and TDC, with 43.8% of TDC (4017 children) living in households with income greater than or equal to 400% of the federal poverty level, compared with only 27.1% of children with CP (41 children).

**Table 1.** Descriptive Characteristics of Participants, by Group

Characteristic	Children, No. (%)		*P* value^a^
	Children with CP (n = 151)	Typically developing children (n = 9177)	
Age, y			
6-11	67 (44.4)	3759 (41.0)	.40
12-17	84 (56.6)	5418 (59)	
Sex			
Male	91 (60.3)	4446 (48.5)	.004
Female	60 (39.7)	4731 (51.5)	
Race/ethnicity			
Non-Hispanic			.53
White	94 (62.3)	6228 (67.9)	
Black	13 (8.6)	684 (7.4)	
Hispanic	20 (13.2)	1064 (11.6)	
Other^b^	24 (15.9)	1201 (13.1)	
Household income, percentage of federal poverty level			
0-199	59 (39.1)	2536 (25.3)	<.001
200-299	32 (21.2)	1445 (15.8)	
300-399	19 (13.2)	1389 (15.1)	
≥400	41 (27.1)	4017 (43.8)	
Frequent and chronic pain in the last 12 mo			
Yes	50 (33.1)	665 (7.3)	<.001
No	101 (66.9)	8474 (92.7)	
Difficulty dressing or bathing			
Yes	83 (56.9)	15 (0.2)	<.001
No	63 (43.1)	9076 (99.8)	
Participation in sport in the last 12 mo			
Yes	43 (30.1)	5998 (66.3)	<.001
No	100 (69.9)	3051 (33.7)	

Abbreviation: CP, cerebral palsy.

^a^ *P* values were calculated with 2-sided χ^2^ tests.

^b^ Other includes Asian, American Indian or Alaska Native, and Native Hawaiian or other Pacific Islander.

Table 2 provides estimates of the associations of independent variables with difficulty in dressing or bathing and participation in sports. After controlling for age, sex, and race, children with CP with pain were 3.03 times more likely to have difficulty in dressing or bathing compared with children with CP without pain (adjusted odds ratio, 3.03; 95% CI, 1.42-6.42), and children with CP with pain were 60% less likely to participate in sports compared with children with CP without pain (adjusted odds ratio, 0.40; 95% CI, 0.35-0.45).

**Table 2.** Estimates of Associations of Independent Variables With Participation in Sports and Difficulty in Dressing or Bathing

Parameter	AOR (95% CI)	
	Participation in sports	Difficulty in dressing or bathing
Dependent variable		
CP		
Yes	0.28 (0.18-0.43)^a^	1102.08 (468.17-2594.26)^a^
No	1 [Reference]	1 [Reference]
Chronic pain		
Yes	0.84 (0.71-0.99)^a^	15.8 (5.64-44.26)^a^
No	1 [Reference]	1 [Reference]
Age	0.95 (0.93-0.96)^a^	0.92 (0.85-1.00)
Sex		
Female	0.60 (0.54-0.65)^a^	0.85 (0.48-1.52)
Male	1 [Reference]	1 [Reference]
Race/ethnicity		
Hispanic	0.73 (0.61-0.87)^a^	2.77 (0.97-7.92)
Non-Hispanic		
White	1.25 (1.09-1.42)^a^	0.98 (0.43-2.22)
Black	0.91 (0.76-1.12)	0.65 (0.18-2.36)
Other^b^	1 [Reference]	1 [Reference]
Interaction		
Children with CP with pain vs without pain	0.40 (0.35-0.45)^a^	3.03 (1.42-6.42)^a^
Typically developing children with pain vs without pain	0.84 (0.71-0.99)^a^	15.81 (5.66-44.18)^a^

Abbreviations: AOR, adjusted odds ratio; CP, cerebral palsy.

^a^ *P* ≤ .05.

^b^ Other includes Asian, American Indian or Alaska Native, and Native Hawaiian or other Pacific Islander.

## Discussion

As expected, difficulty dressing or bathing and low participation in sport were much more common among children with CP than among TDC. A novel finding of this study was that the issue was exacerbated by the presence of pain, which was present in approximately one-third of the children with CP. However, pain also limited participation in sport and was negatively associated with difficulty dressing or bathing among TDC. Although CP is a complicated disorder and the difficulties associated with dressing or bathing and participation in sport are associated with the poor motor control of children with CP, these findings suggest that pain is also a notable contributing factor. A similar study^6^ found that 33% of parents reported that pain had a negative effect on normal activities, specifically among some functional activities similar to dressing or bathing. Because children with CP face many barriers to performing motor skill activities in various settings,^2^ identification and treatment of pain may improve participation. Although the prevalence of pain is often assessed and monitored through surveillance,^1^ clinicians should consider regularly enhanced approaches to identifying and screening for pain.

This study has limitations related to the parent-reported observational nature, and there are always potentially confounding variables. Further research is needed to examine additional confounders and the underlying causes of pain and their association with motor skill activities.

## References

[1] Ostojic K, Paget S, Kyriagis M, Morrow A. Acute and chronic pain in children and adolescents with cerebral palsy: prevalence, interference, and management. Arch Phys Med Rehabil. 2020;101(2):213-219. doi:10.1016/j.apmr.2019.08.475 31521713

[2] Walker A, Colquitt G, Elliott S, Emter M, Li L. Using participatory action research to examine barriers and facilitators to physical activity among rural adolescents with cerebral palsy. Disabil Rehabil. 2020;42(26):3838-3849. doi:10.1080/09638288.2019.1611952 31088164

[3] Van Zelst BR, Miller MD, Russo R, Murchland S, Crotty M. Activities of daily living in children with hemiplegic cerebral palsy: a cross-sectional evaluation using the Assessment of Motor and Process Skills. Dev Med Child Neurol. 2006;48(9):723-727. doi:10.1017/S0012162206001551 16904017

[4] von Elm E, Altman DG, Egger M, Pocock SJ, Gøtzsche PC, Vandenbroucke JP; STROBE Initiative. The Strengthening the Reporting of Observational Studies in Epidemiology (STROBE) statement: guidelines for reporting observational studies. Lancet. 2007;370(9596):1453-1457. doi:10.1016/S0140-6736(07)61602-X 18064739

[5] Child and Adolescent Health Measurement Initiative. 2017-208 National Survey of Children’s Health (2 years combined data set): child and family health measures, national performance and outcome measures, and subgroups, SAS codebook, version 1.0. Published 2019. Accessed December 1, 2020. http://www.childhealthdata.org

[6] Tervo RC, Symons F, Stout J, Novacheck T. Parental report of pain and associated limitations in ambulatory children with cerebral palsy. Arch Phys Med Rehabil. 2006;87(7):928-934. doi:10.1016/j.apmr.2006.02.023 16813780

